# Provision of weight loss programmes and their influence on weight after 1 year: follow-up survey of usual care in the STEPWISE study

**DOI:** 10.1192/bjb.2019.59

**Published:** 2019-10

**Authors:** Lizzie Swaby, Richard Holt, Rebecca Gossage-Worrall, Daniel Hind

**Affiliations:** Study Manager, Clinical Trials Research Unit (CTRU), University of Sheffield. email: e.a.swaby@sheffield.ac.uk; Professor in Diabetes and Endocrinology, University of Southampton; Study Manager, CTRU, University of Sheffield; Assistant Director, CTRU, University of Sheffield

In 2014, the National Institute of Health and Care Excellence (NICE) recommended that people with psychosis, especially those taking antipsychotic medication, should be offered a combined physical activity and healthy eating intervention.^1^ Moreover, from 2014, Commissioning for Quality and Innovation (CQUIN) supported these recommendations by introducing annual financial incentives to mental health trusts in England to improve their provision of physical healthcare.^2^ However, our previous survey conducted in 2015 of ten UK National Health Service mental health trusts participating in the STEPWISE trial^3^ highlighted that such interventions were largely unavailable, and where programmes were offered these were variable and not always widely accessed.^4^

As a follow-up to our previous survey,^4^ and in conjunction with the STEPWISE study,^3^ we investigated changes in usual care provision of physical activity and/or healthy eating programmes during the course of the STEPWISE trial. As with the initial survey, mental health professionals completed the survey on behalf of the same ten participating STEPWISE sites.

The number of trusts offering trust-led lifestyle programmes declined from eight to four out of ten during the study. In those trusts not offering programmes, patients were often signposted to externally run services, such as council or voluntary-sector programmes, which was also the case in the first survey.

Seven sites reported changes in services since the previous survey. These included an increased awareness of the need for physical health monitoring; expansion of existing services such as dietitians, healthy living services and gyms; and new provision of services, for instance, one trust had formed a planning group to develop a trust-led programme.

More trusts reported offering support to stop smoking than in the first survey (60% *v*. 30%), with three trusts either being or becoming smoke free since baseline, and more sites providing access to smoking cessation advisors.

A similar number of surveyed trusts reported recording biomedical measures prior to starting antipsychotic medication, but more trusts reported ongoing monitoring of physical health measures at 1 year than in the first survey, indicating a move towards compliance with the NICE guidelines.

This follow-up survey suggests that trusts are beginning to implement some aspects of NICE physical health recommendations, but the availability of lifestyle programmes offered to patients remains limited. Possibly reflecting the influence of the national CQUIN programme, awareness of the importance of physical health in mental health is increasing, and staff are reportedly more knowledgeable about the services that are available to patients within their trust, including programmes and facilities offered by external services.

Further research would be needed in order to generalise these findings to all UK mental health trusts, as only STEPWISE participating sites were surveyed, which may have resulted in a biased sample. In addition, a longer-term follow-up may have revealed more substantial changes by allowing more time to overcome barriers and implement processes.
